# Disease Milestones through Bibliometric Analysis of the Top 100 Cited Articles in Multiple Myeloma

**DOI:** 10.7759/cureus.2438

**Published:** 2018-04-05

**Authors:** Azka Latif, Vikas Kapoor, Qurat ul ain riaz Sipra, Saad Ullah Malik, Jawad Bilal, Irbaz Bin Riaz, Faiz Anwer

**Affiliations:** 1 Hematology and Oncology, University of Arizona, Tucson, USA; 2 Department of Internal Medicine, University of Arizona, Tucson, USA; 3 Medicine, University of Arizona, Tucson, USA; 4 Hematology and Oncology, Mayo Clinic and Foundation, Rochester, MN, USA

**Keywords:** bibliometeric analysis, multiple myeloma, citation analysis

## Abstract

Multiple myeloma (MM) accounts for 1.6% of all cancers and 5%-10% of all hematologic malignancies in the United States (US). Despite marked progress in disease management, it remains incurable with high rates of relapse. We conducted a bibliographic analysis on the Web of Science (WOS) from July 25, 2017 and July 29, 2017. Among the top 100 most-cited articles (1901-2012), the most cited article received 2404 citations and least cited article received 336 citations. Forty-four of 100 articles were published in journals with impact factors greater than 20. We observed that over the years, the focus of research has shifted from diagnosis, staging, and pathogenesis to better treatment outcomes. A subgroup analysis of the top 100 cited articles published in the last five years (2012-2017) demonstrated that several landmark studies, which will likely change the landscape of treating multiple myeloma, were not included in the top 100 list. Interestingly, most of these articles were focused on novel therapeutic agents. This bibliographic analysis provides a list of the 100 top-cited articles in multiple myeloma along with the captivating comprehension of the history and development in various aspects of disease processes. The landscape of this disease is rapidly evolving, and bibliometric studies such as the one presented provide a valuable tool that can highlight the important transitions in the field.

## Introduction and background

Multiple myeloma (MM) accounts for 1.6% of all cancers and 5% to 10% of all hematologic malignancies in the United States (US) [1]. Worldwide, approximately 154,000 cases are diagnosed, and 101,000 deaths are attributed to MM every year [2]. Due to the advent of novel therapeutic agents, median overall survival has increased from one to two years to seven to eight years with a meaningful improvement in the quality of life [1]. Although there has been marked progress in disease management, MM remains incurable with high rates of relapse. Ongoing clinical trials have significantly contributed to favourable disease outcomes; however, many of these interventions remain unknown to clinicians. This highlights the need for citation analysis to reflect these advances and substantial progress in this field.

Citation analysis is a type of bibliometric analysis in which evaluation and ranking of an article or journal is done on the basis citation count [3]. It identifies the milestones completed in understanding core aspects of a disease and emphasizes on major developments made in the subject matter [4-5]. Clinicians often modify their disease management based on research published in high impact journals [6] thus if most important articles can be highlighted via citation analysis it will help clinicians in making better choices for their patients. So far, no such study has been performed to conclude the most influential articles in the field of MM. The aim of the current study is to identify the 100 top-cited publications in MM and highlight the most significant advances made in the field over the preceding several decades.

## Review

Materials and methods

We conducted a bibliographic analysis on the Web of Science (WOS). The time covered in WOS is between 1900 and 2017. We included journals listed in the Science Citation Index Expanded, without specific restrictions on the journals. We retrieved articles for analysis by typing “Multiple Myeloma” into the WOS search box and conducted data search with the application of English language filter on July 25, 2017. We identified 27,718 articles that were published between 1901 and 2017, ranked the articles based on citation frequency from highest to lowest, and thereafter, shortlisted the top 100 cited articles. Statistical analysis of studies was not performed, and data were reported in the form of tables. To capture the most important and latest research, we conducted a second search on July 29, 2017 to limit articles to those published during the last five years.

Results

Among the top 100 included articles, the most cited article received 2404 citations while the least cited article received 336 citations. All articles were arranged according to citation frequency (Table 1). The top 100 cited articles were published between 1990 and 2007. In our analysis, we found that the highest number of articles were published in the year 2007 (Table 2). Forty-eight of the 100 articles were published in journals with impact factors (IF) greater than 20 (Table 3). The journal with the highest number of publications was Blood with 33% of the publications (IF 13.16) followed by New England Journal of Medicine (NEJM) with 20% of the publications (IF 72.406). The country of origin with the highest number of publications on the topic of MM was the US (n=73) followed by France (n=10), Italy, and Germany (Table 4). These 100 articles sourced from 50 major institutions, with top three most significant contributors being Dana Farber Cancer Institute, Mayo Clinic, and the University of Arkansas Medical Sciences (Table 5). Most articles focused on disease management followed by pathogenesis and disease staging respectively (Table 6).

**Table 1:** Top 100 cited articles on the topic of multiple myeloma

No.	Author	Title	Journal	Number of Citations
1	Durie, BGM et al.	Clinical staging system for multiple-myeloma - correlation of measured myeloma cell mass with presenting clinical features, response to treatment, and survival	CANCER 1975;36(842);1182674;	2404
2	Attal, M	A prospective, randomized trial of autologous bone marrow transplantation and chemotherapy in multiple myeloma	New England journal of medicine 1996 Jul 11;335(2):91-7	1909
3	Singhal, S et al.	Antitumor activity of thalidomide in refractory multiple myeloma.	New England journal of medicine 1999;341(91);10564685	1665
4	Kawano, M et al.	Autocrine generation and requirement of bsf-2/il-6 for human multiple myeloma	Nature 1988;332(83);3258060	1547
5	Richardson, PG et al.	Bortezomib or high-dose dexamethasone for relapsed multiple myeloma	New England journal of medicine 2005;352(2487);15958804	1564
6	Durie, BGM et al.	International uniform response criteria for multiple myeloma	Leukemia 2006;20(1467);16855634	1222
7	Child, JA et al.	High-dose chemotherapy with hematopoietic stem-cell rescue for multiple myeloma	New England journal of medicine 2003;348(1875);12736280	1070
8	Greipp, PR et al.	International staging system for multiple myeloma	Journal of clinical oncology 2005;23(3412);15809451	1064
9	Hideshima, T et al.	The proteasome inhibitor PS-341 inhibits growth, induces apoptosis, and overcomes drug resistance in human multiple myeloma cells	Cancer research 2001;61(3071);11306489	1063
10	Kumar, Shaji et al.	Improved survival in multiple myeloma and the impact of novel therapies	Blood 2008;111(2516);17975015	1053
11	Kyle, RA et al.	Criteria for the classification of monoclonal gammopathies, multiple myeloma and related disorders: a report of the International Myeloma Working Group	British journal of Hematology 2003;121(749);12780789	998
12	San M et al.	Bortezomib plus melphalan and prednisone for initial treatment of multiple myeloma	NEJM 2008;359(906);18753647	983
13	Kyle RA et al.	Drug therapy: Multiple myeloma	NEJM 2004;351(1860);15509819	869
14	Tian, E et al.	The role of the Wnt-signaling antagonist DKK1 in the development of osteolytic lesions in multiple myeloma	NEJM 2003;349(2483);14695408	832
15	Dimopoulos, M et al.	Lenalidomide plus dexamethasone for relapsed or refractory multiple myeloma	NEJM 2007;357(2123);18032762	817
16	Kyle, RA et al.	Review of 1027 patients with newly diagnosed multiple myeloma	Mayo clinic proceedings 2003;78(21);12528874	786
17	Kyle, RA et al.	Multiple-myeloma - review of 869 cases	mayo clinic proceedings 1975;50(29);1110582	779
18	Palumbo, A et al.	Medical progress multiple myeloma	NEJM 2011;364(1046);21410373	750
19	Weber, DM et al.	Lenalidomide plus dexamethasone for relapsed multiple myeloma in North America	NEJM 2007;357(2133);18032763	743
20	Bernson, JR et al.	Efficacy of pamidronate in reducing skeletal events in patients with advanced multiple myeloma	NEJM 1996;334(488);8559201	703
21	Chapman, MA et al.	Initial genome sequencing and analysis of multiple myeloma	Nature 2011;471(467)21430775	671
22	Hideshima, T et al.	NF-kappa B as a therapeutic target in multiple myeloma	journal of biological chemistry 2002;277(16639);11872748	662
23	Attal, M et al.	Single versus double autologous stem-cell transplantation for multiple myeloma	NEJM 2003;349(2495);14695409	644
24	Hideshima, T et al.	Thalidomide and its analogs overcome drug resistance of human multiple myeloma cells to conventional therapy	Blood 2000;96(243);11049970	640
25	Dalton, WS et al.	Drug-resistance in multiple-myeloma and non-Hodgkin’s lymphoma - detection of p-glycoprotein and potential circumvention by addition of verapamil to chemotherapy	Journal of clinical oncology 1989;7(415);2564428	587
26	Davies, FE et al.	Thalidomide and immunomodulatory derivatives augment natural killer cell cytotoxicity in multiple myeloma	Blood 2001;98(210);11418482	586
27	Barlogie, B et al.	Effective treatment of advanced multiple-myeloma refractory to alkylating-agents	NEJM 1984;310(1353);6546971	586
28	Keats, JJ et al.	Promiscuous mutations activate the noncanonical NF-kappa B pathway in multiple myeloma	Cancer Cell 2007;12(131);17692805	580
29	Richardson, PG et al.	Immunomodulatory drug CC-5013 overcomes drug resistance and is well tolerated in patients with relapsed multiple myeloma	Blood 2002;100(3063);12384400	567
30	Hallek, M et al.	Multiple myeloma: Increasing evidence for a multistep transformation process	Blood 1998;91(3);9414264	565
31	Annunziata, CM et al.	Frequent engagement of the classical and alternative NF-kappa B pathways by diverse genetic abnormalities in multiple myeloma	Cancer Cell 2007;12(115);17692804	552
32	Rajkumar, SV et al.	Phase III clinical trial of thalidomide plus dexamethasone compared with dexamethasone alone in newly diagnosed multiple myeloma: A clinical trial coordinated by the eastern cooperative oncology group	Journal of clinical oncology 2006;24(431);16365178	551
33	Palumbo, A et al.	Oral melphalan and prednisone chemotherapy plus thalidomide compared with melphalan and prednisone alone in elderly patients with multiple myeloma: randomised controlled-trial	Lancet 2006;367(825);16530576	549
34	Rosen, LS et al.	Zoledronic acid versus pamidronate in the treatment of skeletal metastases in patients with breast cancer or osteolytic lesions of multiple myeloma: A phase III, double-blind, comparative trial	Cancer Journal 2001;7(377);11693896	530
35	Facon, T et al.	Melphalan and prednisone plus thalidomide versus melphalan and prednisone alone or reduced-intensity autologous stem cell transplantation in elderly patients with multiple myeloma (IFM 99-06): a randomised trial	Lancet 2007;370(1209);17920916	529
36	Mitsiades, N et al.	Molecular sequelae of proteasome inhibition in human multiple myeloma cells	Proceedings of the national academy of sciences of USA 2002;99(14374);12391322	514
37	Mitsiades, N et al.	The proteasome inhibitor PS-341 potentiates sensitivity of multiple myeloma cells to conventional chemotherapeutic agents: therapeutic applications	Blood 2003;101(2377);12424198	505
38	Peterson,TR et al.	DEPTOR Is an mTOR Inhibitor Frequently Overexpressed in Multiple Myeloma Cells and Required for Their Survival	Cell 2009;137(873);19446321	504
39	Zhan, F et al.	The molecular classification of multiple myeloma	Blood 2006;108(2020);16728703	502
40	Rosen, LS et al.	Long-term efficacy and safety of zoledronic acid compared with pamidronate disodium in the treatment of skeletal complications in patients with advanced multiple myeloma or breast carcinoma - A randomized, double-blind, multicentre, comparative trial	Cancer 2003;98(1735);14534891	493
41	Alexanian, R et al.	Treatment for multiple myeloma - combination chemotherapy with different melphalan dose regimens	Journal of American Medical Association 1969;208(1689);5818682	493
42	Chesi, M et al.	Frequent translocation t(4;14) (p16.3; q32.3) in multiple myeloma is associated with increased expression and activating mutations of fibroblast growth factor receptor 3	Nature Genetics 1997;16(260);9207791	491
43	Gutterman, JU et al.	Leukocyte interferon-induced tumor-regression in human metastatic breast-cancer, multiple-myeloma, and malignant-lymphoma	Annals of Internal Medicine 1980;93(399);6159812	489
44	Barlogie, B et al.	Thalidomide and hematopoietic-cell transplantation for multiple myeloma	NEJM 2006;354(1021);16525139	487
45	Kuehl, WM et al.	Multiple myeloma: Evolving genetic events and host interactions	Nature Reviews Cancer 2002;2(175);11990854	486
46	Obeng, EA et al.	Proteasome inhibitors induce a terminal unfolded protein response in multiple myeloma cells	Blood 2006;107(4907);16507771	483
47	Matsui, W et al.	Characterization of clonogenic multiple myeloma cells	Blood 2004;103(2332);14630803	477
48	Rajkumar, SV et al.	Lenalidomide plus high-dose dexamethasone versus lenalidomide plus low-dose dexamethasone as initial therapy for newly diagnosed multiple myeloma: an open-label randomised controlled trial	Lancet Oncology 2010;11(29);19853510	474
49	Vacca, A et al.	Bone-marrow angiogenesis and progression in multiple-myeloma	British Journal of Hematology 1994;87(503);7527645	474
50	Kyle, RA et al.	Criteria for diagnosis, staging, risk stratification and response assessment of multiple myeloma	Leukemia 2009;23(3);18971951	472
51	Chauhan, D et al.	A novel orally active proteasome inhibitor induces apoptosis in multiple myeloma cells with mechanisms distinct from Bortezomib	Cancer Cell 2005;8(407);16286248	472
52	Vacca, A et al.	Bone marrow neovascularization, plasma cell angiogenic potential, and matrix metalloproteinase-2 secretion parallel progression of human multiple myeloma	Blood 1999;93(3064);10216103	467
53	Bharti, AC et al.	Curcumin (diferuloylmethane) down-regulates the constitutive activation of nuclear factor-kappa B and I kappa B alpha kinase in human multiple myeloma cells, leading to suppression of proliferation and induction of apoptosis	Blood 2003;101(1053);12393461	458
54	Mitsiades, N et al.	Apoptotic signalling induced by immunomodulatory thalidomide analogs in human multiple myeloma cells: therapeutic implications	Blood 2002;99(4525);12036884	458
55	Avet, LH et al.	Genetic abnormalities and survival in multiple myeloma: the experience of the Intergroupe Francophone du Myelome	Blood 2007;109(3489);17209057	453
56	Mitsiades, CS et al.	Inhibition of the insulin-like growth factor receptor-1 tyrosine kinase activity as a therapeutic strategy for multiple myeloma, other hematologic malignancies, and solid tumors	Cancer Cell 2004;5(221);15050914	449
57	Klein, B et al.	Interleukin-6 in human multiple-myeloma	Blood 1995;85(863);7849308	449
58	Hideshima, T et al.	Understanding multiple myeloma pathogenesis in the bone marrow to identify new therapeutic targets	Nature Reviews Cancer 2007;7(585);17646864	447
59	Barlogie, B et al.	Total therapy with tandem transplants for newly diagnosed multiple myeloma	Blood 1999;9355);9864146	442
60	Broder, S et al.	Impaired synthesis of polyclonal (non-paraprotein) immunoglobulins by circulating lymphocytes from patients with multiple-myeloma - role of suppressor cells	NEJM 1975;293(887);1080834	442
61	McCarthy, PL et al.	Lenalidomide after Stem-Cell Transplantation for Multiple Myeloma	NEJM 2012;366(1770);22571201	441
62	Zhan, F et al.	Global gene expression profiling of multiple myeloma, monoclonal gammopathy of undetermined significance, and normal bone marrow plasma cells	Blood 2002;99(1745);11861292	436
63	Ludwig, H et al.	Erythropoietin treatment of anemia associated with multiple-myeloma	NEJM 1990;322(1693);2342535	434
64	Kunzmann, V et al.	Stimulation of gamma delta T cells by aminobisphosphonates and induction of antiplasma cell activity in multiple myeloma	Blood 2000;96(384);10887096	433
65	Henry, DH et al.	Randomized, Double-Blind Study of Denosumab Versus Zoledronic Acid in the Treatment of Bone Metastases in Patients with Advanced Cancer (Excluding Breast and Prostate Cancer) or Multiple Myeloma	Journal of Clinical Oncology 2011;29(1125);21343556	428
66	Attal, M et al.	Maintenance, therapy with thalidomide improves survival in patients with multiple myeloma	Blood 2006;108(3289);16873668	426
67	Barlogie, B et al.	Superiority of tandem autologous transplantation over standard therapy for previously untreated multiple myeloma	Blood 1997;89(789);9028309	425
68	Hideshima, T et al.	Advances in biology of multiple myeloma: clinical applications	Blood 2004;104(607);15090448	424
69	Orlowski, RZ et al.	Randomized phase III study of PEGylated liposomal doxorubicin plus bortezomib compared with bortezomib alone in relapsed or refractory multiple myeloma: Combination therapy improves time to progression	Journal of Clinical Oncology 2007;25(3892);17679727	423
70	Shaughnessy, JD et al.	A validated gene expression model of high-risk multiple myeloma is defined by deregulated expression of genes mapping to chromosome 1	Blood 2007;109(2276);17105813	422
71	Berenson, JR et al.	Long-term pamidronate treatment of advanced multiple myeloma patients reduces skeletal events	Journal of Clinical Oncology 1998;16(593);9469347	419
72	Richardson, PG et al.	Lenalidomide, bortezomib, and dexamethasone combination therapy in patients with newly diagnosed multiple myeloma	Blood 2010;116(679);20385792	416
73	Attal, M et al.	Lenalidomide Maintenance after Stem-Cell Transplantation for Multiple Myeloma	NEJM 2012;366(1782);22571202	411
74	Mitsiades, CS et al.	Transcriptional signature of histone deacetylase inhibition in multiple myeloma: Biological and clinical implications	proceeding of the national academy of sciences of the united states of America 2004;101(540);14695887	404
75	Chauhan, D et al.	Multiple myeloma cell adhesion-induced interleukin-6 expression in bone marrow stromal cells involves activation of NF-kappa B	Blood 1996;87(1104);8562936	403
76	Cavo, M et al.	Bortezomib with thalidomide plus dexamethasone compared with thalidomide plus dexamethasone as induction therapy before, and consolidation therapy after, double autologous stem-cell transplantation in newly diagnosed multiple myeloma: a randomised phase 3 study	Lancet 2010;376(2075);21146205	402
77	Dankbar, B et al.	Vascular endothelial growth factor and interleukin-6 in paracrine tumor-stromal cell interactions in multiple myeloma	Blood 2000;95(2630);10753844	396
78	Retting, MB et al.	Kaposi's sarcoma-associated herpesvirus infection of bone marrow dendritic cells from multiple myeloma patients	Science 1997;276(1851);9188529	396
79	Weber, DM et al.	Thalidomide alone or with dexamethasone for previously untreated multiple myeloma	Journal of Clinical Oncology 2003;21(16);12506164	394
80	Moreau, P et al.	Subcutaneous versus intravenous administration of bortezomib in patients with relapsed multiple myeloma: a randomised, phase 3, non-inferiority study	Lancet Oncology 2011;12(431);21507715	392
81	Barlogie, B et al.	Extended survival in advanced and refractory multiple myeloma after single-agent thalidomide: identification of prognostic factors in a phase 2 study of 169 patients	Blood 2001;98(492);11435324	391
82	Kyle RA et al.	Multiple myeloma	Blood 2008;111(2962);18332230	386
83	Tricot, G et al.	Peripheral-blood stem-cell transplants for multiple-myeloma - identification of favourable variables for rapid engraftment in 225 patients	Blood 1995;85(588);7529066	383
84	Bataille, R et al.	Multiple myeloma	NEJM 1997;336(1657);9171069	375
85	Fonseca, R et al.	International Myeloma Working Group molecular classification of multiple myeloma: spotlight review	Leukemia 2009;23(2210);19798094	372
86	Hideshima, T et al.	Small-molecule inhibition of proteasome and aggresome function induces synergistic antitumor activity in multiple myeloma	proceedings of the national academy of sciences of the united states of America 2005;102(8567);15937109	372
87	Sonneveld, P et al.	Modulation of multidrug-resistant multiple-myeloma by cyclosporine	Lancet 1992;340(255);1353189	362
88	Raab, MS et al.	Multiple myeloma	Lancet 2009;374(324);19541364	357
89	Mitsiades, CS et al.	Activation of NF-kappa B and upregulation of intracellular anti-apoptotic proteins via the IGF-1/Akt signalling in human multiple myeloma cells: therapeutic implications	Oncogene 2002;21(5673);12173037	357
90	Loeffler, D et al.	Interleukin-6-dependent survival of multiple myeloma cells involves the Stat3-mediated induction of microRNA-21 through a highly conserved enhancer	Blood 2007;110(1330);17496199	355
91	Bergsagel, PL et al.	Cyclin D dysregulation: an early and unifying pathogenic event in multiple myeloma	Blood 2005;106(296);15755896	355
92	DiPersio, JF et al.	Plerixafor and G-CSF versus placebo and G-CSF to mobilize hematopoietic stem cells for autologous stem cell transplantation in patients with multiple myeloma	Blood 200;113(5720);19363221	354
93	Mandelli, F et al.	Maintenance treatment with recombinant interferon alfa-2b in patients with multiple-myeloma responding to conventional induction chemotherapy	NEJM 190;322(1430);2184356	352
94	Landgren, O et al.	Monoclonal gammopathy of undetermined significance (MGUS) consistently precedes multiple myeloma: a prospective study	Blood 2009;113(5412);19179464	349
95	Pasquali, S et al.	Combination chemotherapy versus melphalan plus prednisone as treatment for multiple myeloma: An overview of 6,633 patients from 27 randomized trials	Journal of Clinical Oncology 1998;16(3832);9850028	348
96	Fermand, JP et al.	High-dose therapy and autologous peripheral blood stem cell transplantation in multiple myeloma: Up-front or rescue treatment? Results of a multicentre sequential randomized clinical trial	Blood 1998;92(3131);9787148	342
97	Richardson, PG et al.	A randomized phase 2 study of lenalidomide therapy for patients with relapsed or relapsed and refractory multiple myeloma	Blood 2006;108(3458);16840727	340
98	Gupta, D et al.	Adherence of multiple myeloma cells to bone marrow stromal cells upregulates vascular endothelial growth factor secretion: therapeutic applications	Leukemia 2001;15(1950);11753617	340
99	Richardson, PG et al.	Frequency, characteristics, and reversibility of peripheral neuropathy during treatment of advanced multiple myeloma with bortezomib	Journal of Clinical Oncology 2006;24(3113);16754936	338
100	Dispenzieri, A et al.	International Myeloma Working Group guidelines for serum-free light chain analysis in multiple myeloma and related disorders	Leukemia 2009;23(215);19020545	336

**Table 2:** Distribution of articles by year of publication

Publication Year	Number of Records
2007	10
2006	9
2003	9
2009	7
2002	7
2005	5
2004	5
2001	5
2011	4
1998	4
1997	4
2010	3
2008	3
2000	3
1999	3
1996	3
1975	3
2012	2
1995	2
1990	2
1994	1
1992	1
1989	1
1988	1
1984	1
1980	1
1969	1

**Table 3:** Journals in which Top 100 cited articles were published

Source Journals	Impact Factor	Number of Records
Blood	13.16	33
New England journal of medicine	72.406	20
Journal of Clinical Oncology	24.008	9
Leukemia	11.702	5
The LANCET	47.83	5
Cancer cell	27.4	4
Proceedings of The National Academy of Sciences of The United States of America	9.661	3
Nature reviews cancer	37.147	2
Nature	40.137	2
Mayo Clinic Proceedings	6.686	2
LANCET Oncology	33.9	2
Cancer	5.99	2
British Journal of Haematology	5.67	2
Cancer Research	9.122	1
Journal of Biological Chemistry	4.125	1
Cancer Journal	4.218	1
Cell	30.41	1
Journal of American Medical Association	44.405	1
Nature Genetics	27.959	1
Annals of Internal Medicine	17.202	1
Science	37.205	1
Oncogene	7.519	1

**Table 4:** Country of origin for top 100 cited articles

Countries	Number of Records
USA	73
France	10
Italy	7
Germany	4
England	1
Spain	1
Netherlands	1
Austria	1
Japan	1
Greece	1

**Table 5:** Institutions contributing in the number of publications

Institutions	Number of Records
Dana Farber Cancer Institute	22
Mayo Clinic	13
University of Arkansas Medical Sciences	9
UTMD Anderson Cancer Center	5
University of California Los Angeles	4
Chu de Toulouse	4
Chu de Nantes	3
NIH national cancer institute (NCI)	3
University of Turin	2
University of Bari Bari	2
University of Arizona	2
John Hopkins University	1
Eli & Etdythe l. Broad Institute, Seven Cambridge Centers	1
University of Munich	1
Cancer Institute Medical Group	1
Adult division of The South West Cancer Chemotherapy Study Group	1
University of Miami Miller School of Medicine	1
University of South Carolina	1
Hiroshima University	1
Whitehead Institute Biomedical Research, Nine Cambridge Center	1
Bethesda Naval Hospital, Center Cancer Research	1
University of Leeds	1
International Myeloma Working Group	1
University of Athens School of Medicine	1
National Institute of Health	1
Chu Lille	1
University of Salamanca	1
University of Bologna	1
Sapienza University Rome	1
Erasmus University Rotterdam	1
Cedars Sinai Outpatient Cancer Center	1
University of Wurzburg	1
University of Muenster	1
Washington University	1
St Louis Hospital	1
University of North Carolina	1
University of Vienna	1
Institute of Molecular Genetics	1
Roswell Park Center Institute	1
University of Leipzig	1
Joan Karnell Cancer Center	1
Arcispedale Santa Maria Nuova	1

**Table 6:** Classification of articles by categories.

Category	Number of Studies
Management	51
Pathogenesis	33
Staging	3
Review Articles	4

Regarding authors with the highest number of publications, Hideshima T and Mitsiades CS ranked first with six publications each, followed by Barlogie B, Kyle RA, and Richardson PG with five publications each, and Attal M with four publications (Table 7). Anderson KC was the top author with 26 publications as co-author. Most of the articles were categorized under the title of Hematology (40%) followed by General Internal Medicine (29%), and Oncology (27%), respectively.

**Table 7:** Most common first 15 authors

Author Name	Number of Records
Hideshima T	6
Mitsiades CS	6
Richardson PG	5
Kyle RA	5
Barlogie B	5
Attal M	4
Durie BGM	2
Chauhan D	2
Rajkumar SV	2
Palumbo A	2
Rosen LS	2
Bernson JR	2
Vacca A	2
Weber DM	2
Singhal S	1

A subgroup analysis was performed to capture the development and progress of MM therapy during the last five years. It demonstrated that the most cited article received 441 citations while the least cited article received only 70 citations. Forty-four of the 100 articles were published in 2012, 26 in 2013, and 20 in 2014. The top three journals targeted by authors were Blood (35%), Journal of Clinical Oncology (11%), and NEJM (11%). The author with the most publications as the first author was Palumbo A with five publications, whereas the second position was shared by San-Miguel J, Kumar S, and Richardson PG with four publications each. The country with the highest output in last five years was the US (79%). The top three research areas focused by authors were Hematology (50%), Oncology (38%), and General Internal Medicine (14%).

Discussion

Bibliometric analysis has been used in the past to identify frontiers in specific fields and to evaluate the contribution of authors, institutions, and nations. The total number of citations received by an article represents its overall contribution to the clinical world.

Our study demonstrates that over the years, the focus of research has shifted from diagnosis, staging, and pathogenesis to better treatment outcomes in patients with MM (51 publications). The timeline for the evolution of MM therapy has progressed starting with melphalan-prednisone in 1960’s which was the standard of care for about 30 years. During the next 30 years, therapy further evolved when drugs such as vincristine, doxorubicin, and dexamethasone (VAD), alkylating agents such as Carmustine (VBAD), cyclophosphamide and melphalan (VCMP) were introduced. However, these therapeutic agents did not significantly improve the outcomes. High-dose melphalan followed by autologous stem cell transplant (ASCT) was a step towards favorable clinical outcomes. The armamentarium against MM was revolutionized by the development of ground-breaking agents such as immunomodulators (thalidomide and lenalidomide) and the proteasome inhibitor (bortezomib).

After better treatment outcomes, the most frequently encompassed category was disease pathogenesis (29 publications). Over the years, a thorough understanding of aetiological factors and relation of genetic aberrations to pathogenesis has laid the foundation for significant improvement in disease management and prognostication. Two of the top ten most cited articles were aimed at the staging of disease. The first being the Clinical Staging System proposed by Durie BGM et al., although the most cited article in our list is no longer the primary staging system. Modern-day physicians rely on the International Staging System (eighth most cited article) and cytogenetics to classify MM.

The findings of this analysis demonstrated that 32 of 100 articles were published in general medical journals, for which there may be several reasons. Firstly, general medicine journals capture a wide range of population compared to speciality journals. Secondly, patients with MM are usually co-managed by internists and oncologists which would make the general medicine audience more interested in advancements in MM. Lastly, the novel therapeutic options have different mechanisms of actions and extensive side effect profiles. It is very important for the general internist to be aware of these side effects to effectively manage these patients in both inpatient and outpatient settings.

The authors of these studies targeted high impact factor journals which is evidenced by the fact that most of the articles were published in journals with impact factors greater than 20. This suggests that MM researchers tend to publish in prestigious and well-respected journals that capture a wide range of the population. We noted diversity amongst the authors, as only a total of 12 articles were contributed by the top two publishers as first authors. These findings suggest a diverse group of researchers involved in the MM field.

Among the top 100 cited articles, only seven studies were focused on bortezomib-containing regimens, whereas none of them included carfilzomib or ixazomib based novel therapeutic regimens. This shows that articles with a high frequency of citations consisted mostly of early-published articles. Therefore, one limitation of such articles is that they favour older studies. Among the top 100 list, only two articles from 2012 were included and the articles published after 2012 did not have enough citations to be included in top 100 list. Therefore, we conducted a subgroup analysis of top 100 articles published after 2011. A bibliographic analysis of top cited articles published in the last five years (2012-2017) showed different results from our original search. Only two studies from the sub group analysis were included in the primary analysis due to a lower number of total citations received. Studies 2012, and onwards were focused on latest developments in the field of MM including therapeutic agents such as novel proteasome inhibitors (carfilzomib, ixazomib), monoclonal antibodies (daratumumab, elotuzumab), and chimeric antigen receptor T cell therapy [7-11].

Our primary limitation was conducting the search in the “title mode”. Therefore, articles that did not contain MM in the title were not retrieved or included in our study. Secondly, our search was limited to the WOS database which excludes citations of textbooks and other databases which are weaker at tracking older publications. Finally, articles published in languages other than English were excluded.

## Conclusions

This bibliographic analysis provides a list of the 100 top-cited articles in MM along with the captivating comprehension of the history and development in various aspects of disease processes. The landscape of MM is rapidly evolving, and bibliometric studies such as the one we present provides a valuable tool that can highlight important transitions in the field. As new evidence continues to emerge, these types of analyses can provide a quantitative instrument to guide the researchers and funding agencies to assess the overall direction of the field with limited health care resources.

## References

[1] Michels TC, Petersen KE (2017). Multiple myeloma: diagnosis and treatment. American family physician.

[2] Fitzmaurice C, Allen C, Barber RM (2017). Global, regional, and national cancer incidence, mortality, years of life lost, years lived with disability, and disability-adjusted life-years for 32 cancer groups, 1990 to 2015: a systematic analysis for the global burden of disease study. JAMA Oncol.

[3] Pilkington A (2009). Bibliometrics at Royal Holloway. Enterprise Engineering.

[4] Miao Y, Liu R, Pu Y, Yin L (2017). Trends in esophageal and esophagogastric junction cancer research from 2007 to 2016: a bibliometric analysis. Medicine.

[5] Powell AG, Hughes DL, Wheat JR, Lewis WG (2016). The 100 most influential manuscripts in gastric cancer: a bibliometric analysis. Int J Surg.

[6] Sackett DL, Rosenberg WM, Gray JA, Haynes RB, Richardson WS (1996). Evidence based medicine: what it is and what it isn't. BMJ.

[7] Stewart AK, Rajkumar SV, Dimopoulos MA (2015). Carfilzomib, lenalidomide, and dexamethasone for relapsed multiple myeloma. N Engl J Med.

[8] Lokhorst HM, Plesner T, Laubach JP (2015). Targeting CD38 with daratumumab monotherapy in multiple myeloma. N Engl J Med.

[9] Lonial S, Vij R, Harousseau JL, Facon T (2012). Elotuzumab in combination with lenalidomide and low-dose dexamethasone in relapsed or refractory multiple myeloma. J Clin Oncol.

[10] Zonder JA, Mohrbacher AF, Singhal S (2012). A phase 1, multicenter, open-label, dose escalation study of elotuzumab in patients with advanced multiple myeloma. Blood.

[11] Garfall AL, Maus MV, Hwang WT (2015). Chimeric antigen receptor T cells against CD19 for multiple myeloma. N Engl J Med.

